# *Apple Latent Spherical Virus* Vector as Vaccine for the Prevention and Treatment of Mosaic Diseases in Pea, Broad Bean, and Eustoma Plants by *Bean Yellow Mosaic Virus*

**DOI:** 10.3390/v6114242

**Published:** 2014-11-07

**Authors:** Nozomi Satoh, Tatsuya Kon, Noriko Yamagishi, Tsubasa Takahashi, Tomohide Natsuaki, Nobuyuki Yoshikawa

**Affiliations:** 1 Faculty of Agriculture, Iwate University, Morioka 020-8550, Japan; E-Mails: a2514010@iwate-u.ac.jp (N.S.); tkon@iwate-u.ac.jp (T.K.); nyama@iwate-u.ac.jp (N.Y.); tsubasa-t@imic.co.jp (T.T.); 2 Faculty of Agriculture, Utsunomiya University, Utsunomiya 321-8505, Japan; E-Mail: natsuaki@cc.utsunomiya-u.ac.jp

**Keywords:** ALSV vaccine, pea, broad bean, eustoma, BYMV, preventive and curative effects

## Abstract

We investigated the protective effects of a viral vector based on an *Apple latent spherical viru*s (ALSV) harboring a segment of the *Bean yellow mosaic virus* (BYMV) genome against mosaic diseases in pea, broad bean, and eustoma plants caused by BYMV infection. In pea plants pre-inoculated with the ALSV vaccine and challenge inoculated with BYMV expressing green fluorescence protein, BYMV multiplication occurred in inoculated leaves, but was markedly inhibited in the upper leaves. No mosaic symptoms due to BYMV infection were observed in the challenged plants pre-inoculated with the ALSV vaccine. Simultaneous inoculation with the ALSV vaccine and BYMV also prevented mosaic symptoms in broad bean and eustoma plants, and BYMV accumulation was strongly inhibited in the upper leaves of plants treated with the ALSV vaccine. Pea and eustoma plants were pre-inoculated with BYMV followed by inoculation with the ALSV vaccine to investigate the curative effects of the ALSV vaccine. In both plant species, recovery from mosaic symptoms was observed in upper leaves and BYMV accumulation was inhibited in leaves developing post-ALSV vaccination. These results show that ALSV vaccination not only prevents mosaic diseases in pea, broad bean, and eustoma, but that it is also effective in curing these diseases.

## 1. Introduction

*Apple latent spherical virus* (ALSV), classified into the genus *Cheravirus*, is a small spherical virus approximately 25 nm in diameter, and comprised of a bisegmented, single-stranded RNA genome (RNA1 and RNA2) and three types of coat proteins (Vp25, Vp20, and Vp24) [1,2]. ALSV is normally isolated from apples, but under experimental conditions, it has a broad host range that includes plants from the *Nicotiana* genus (*N. tabacum*, *N. benthamiana*, *N. occidentalis*, and *N. glutinosa*), tomatoes, potatoes of the *Solanaceae* family, and members of the *Cucurbitaceae* family (cucumbers, melons, zucchinis, and sponge gourds), the *Fabaceae* family (soy, adzukis, peas, and broad beans), and the *Rosaceae* family (apples, pears, Japanese pears, peaches, and plums) [1,3]. Among the various plant hosts, ALSV causes clear mosaic symptoms in *Chenopodium quinoa*, but infection is latent in other hosts. Taking advantage of the broad host range and latent infection of ALSV, we investigated the use of ALSV as a viral vector for the expression of foreign genes and the suppression of endogenous genes [4]. ALSV can be used for functional analysis of plant genes because it induces stable RNA silencing (virus-induced gene silencing, VIGS) in different plant species [3,5,6]. This is likely because ALSV does not encode for a strong silencing suppressor protein [7] and it can also invade the meristem tissue and leaf primordium, so that it induces uniform silencing [5,8].

The mechanism of RNA interference-mediated gene silencing is conserved universally across eukaryotes [9]. In plants, which lack an immune system like animals, RNA silencing plays an essential role in defense mechanisms against molecular parasites, such as viruses [10,11,12,13,14]. When a virus infects a host cell, double-stranded viral RNA is synthesized by viral RNA-dependent RNA polymerases and may then be cleaved by Dicer-like proteins into small interfering RNAs (siRNAs), 21–25 nucleotides in length. siRNAs assemble into RNA-induced silencing complexes (RISCs), which in turn target and cleave RNAs with complementary nucleotide sequences. RNA silencing spreads systemically, and may be likened to the immune system of animals [15,16,17,18].

We hypothesized that ALSV vectors may serve as effective vaccines for the control of plant viral diseases by inducing VIGS. What this means is that if a plant is infected in advance with an ALSV vector containing the genomic sequence of the pathogenic target virus (which we refer to here as an ALSV vaccine) in order to induce VIGS, then RNA silencing should be established before the plant is infected with the target pathogenic virus [19,20]. Experiments have been reported in which plants were pre-inoculated with ALSV vaccines harboring a part of the genomic sequences of *Zucchini yellow mosaic virus* (ZYMV) and *Cucumber mosaic virus* (CMV), both of which are pathogenic to *Cucurbitaceae*, and of three species of tospovirus. These plants showed strong interference against challenge inoculation with the corresponding pathogenic viruses, and viral multiplication was strongly inhibited [8,21]. When ZYMV-infected cucumber plants showing mosaic symptoms were inoculated with an ALSV vaccine harboring part of the ZYMV genome, mosaic symptoms did not appear in new upper leaves and normal plant development was restored, indicating that the ALSV vaccine also showed a curative effect [8]. This curative effect was the result of ALSV invasion of shoot apical meristem tissues, which ZYMV is unable to invade, and the defense system against ZYMV was formed in tissues once they were infected with ALSV [8].

*Bean yellow mosaic virus* (BYMV) is a filamentous virus that is approximately 750 nm long. Like ZYMV, it is a member of the genus *Potyvirus*, which is one of the largest genera of plant RNA viruses including ~180 species [22]. BYMV has a relatively wide host range [23,24], and numerous BYMY strains have been isolated [24,25,26]. The wide host range likely results from readily occurring mutations in the viral genome, thus enabling viral proteins to productively interact with host elf4e transcription initiation factors from different plant species with different sequences [27,28]. In Japan, BYMV is the cause of many plant diseases, such as mosaic diseases in adzukis, kidney beans, peas, soybeans, broad beans, spinaches, eustomas, freesias, iris, and gentians [29]. In legumes, BYMV not only causes mosaic and dwarf symptoms in the parts above ground, but also affects the root nodules, causing reduced yields [30,31].

We previously reported that the ALSV vaccines ALSV-BY:P3(+) and ALSV-BY:P3(−), constructed by inserting a 240-bp part of the P3 region of the BYMV genome, showed interference effects against BYMV and strongly suppressed BYMV replication in the experimental plant *N. benthamiana* [8]. Here, we investigated whether the interference effects of ALSV vaccines found in *N. benthamiana* can also be induced in pea, broad bean, and eustoma plants and whether ALSV vaccines can be used to prevent or cure mosaic diseases caused by BYMV.

## 2. Results

### 2.1. Effects of the ALSV Vaccine in Preventing Pea Mosaic Disease

**Figure 1:**
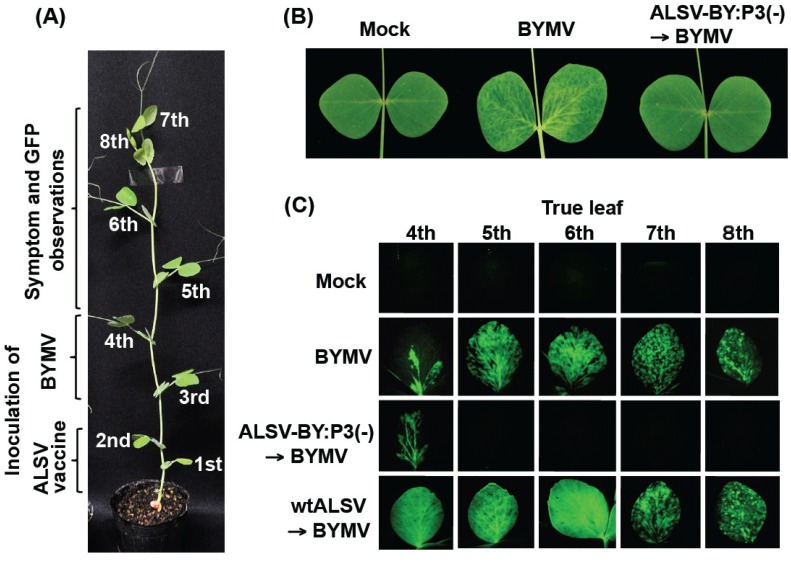
Protective effects of pre-inoculating pea plants with an *Apple latent spherical virus* (ALSV) vaccine (ALSV-BY:P3(−)) on challenge inoculation with a recombinant *Bean yellow mosaic virus* (BYMV) expressing green fluorescent protein (GFP). (**A**) Leaf positions on a pea plant for inoculation with ALSV vaccine and BYMV and observation of symptoms and GFP fluorescence. Pea seedlings (three-leaf stage) were pre-inoculated with the vaccine by rub-inoculating a concentrated ALSV preparation (10 µL per leaf) on the first and second leaf and a crude BYMV extract of IbG-GFP-inoculated *N. benthamiana* was used for challenge inoculations by the carborundum method on the third and fourth leaves of seedlings that had grown to the five-leaf stage; (**B**) Leaf symptoms due to BYMV infection (seventh true leaf) of pea plants inoculated with BYMV (BYMV image) or preinoculation with ALSV-BY:P3(−) followed by challenge inoculation with BYMV (ALSV-BY:P3(−)→BYMV), at 14 days post-inoculation; (**C**) Fluorescence microscopy of the fourth to eighth true leaves of pea plants pre-inoculated with ALSV-BY:P3(−) or wtALSV, followed by challenge inoculation with BYMV 14 dpi.

In pea plants inoculated only with BYMV (*i.e.*, BYMV group; no primary ALSV vaccine inoculation), the fourth leaf showed faint mosaic symptoms at four days post-inoculation (dpi). At 14 dpi, mosaic symptoms were observed from the fifth to the eighth leaves (Figure 1B). In plants that were pre-inoculated with wtALSV followed by BYMV challenge inoculation (wtALSV→BYMV group), the same mosaic symptoms were observed in the fifth to the eighth leaves. However, in the plant group that was pre-inoculated with the ALSV vaccine followed by BYMV challenge inoculation (ALSV-BY:P3(−)→BYMV group), faint mosaic symptoms were observed on the fourth leaf at 4 dpi of BYMV, but no mosaic disease was observed on any upper leaves above the fifth leaf (Figure 1B). The plants in this group did not show any obvious symptoms on their leaves during these experiments, in contrast with BYMV group and the wtALSV→BYMV group, which showed mosaic symptoms on nearly all of their leaves.

Fluorescence from green fluorescence protein (GFP) expression in the fourth to eighth leaves was examined at 14 dpi of BYMV. In the BYMV and wtALSV→BYMV groups, GFP fluorescence was detected in all leaves, and strong fluorescence was observed in particular in the fifth and sixth leaves, which developed after BYMV inoculation. In the ALSV-BY:P3(−)→BYMV group, however, GFP fluorescence was observed in the fourth leaf, but no fluorescence was observed on any upper leaves above the fifth leaf (Figure 1C).

**Figure 2:**
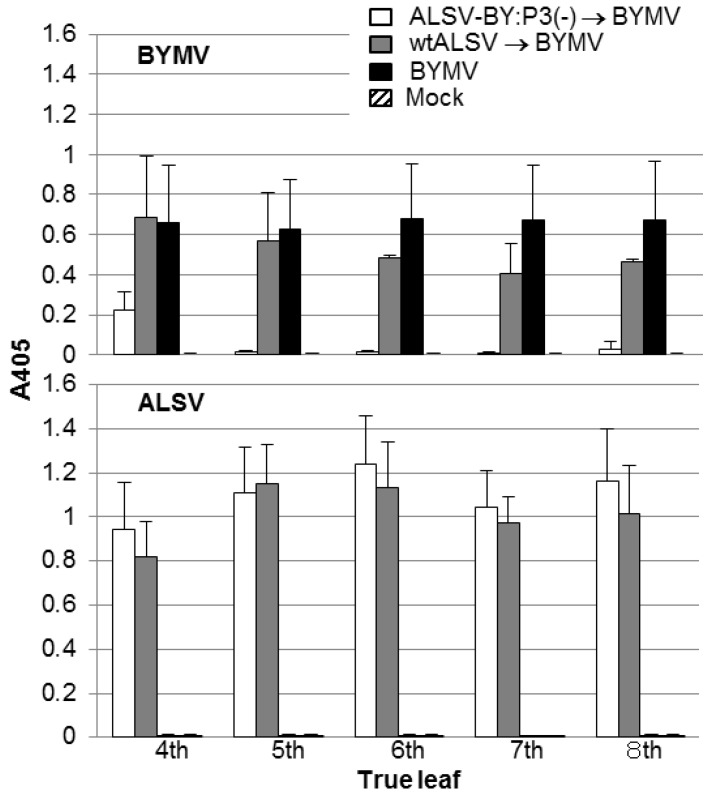
Enzyme-linked immunosorbent assay measuring the accumulation of *Bean yellow mosaic virus* (BYMV) and *Apple latent spherical virus* (ALSV) in pea leaves pre-inoculated with ALSV vaccine (ALSV-BY:P3(−) or wtALSV, followed by challenge inoculation with BYMV-GFP at 14 days post-inoculation. The experimental BYMV series BYMV shows plants not pre-inoculated with ALSV vaccine.

BYMV production was quantified by enzyme-linked immunosorbent assays (ELISAs) at 14 dpi. BYMV was detected in all leaves from the fourth to the eighth leaves in the BYMV and wtALSV→BYMV groups. In the ALSV-BY:P3(−)→BYMV group, however, BYMV was found in the 4th leaf at approximately one-third the level found in the other inoculation groups and was scarcely detected in upper leaves above the fifth leaf (Figure 2). It was frequently observed that BYMV accumulation in wtALSV→BYMV group was lower than that in BYMV group (Figure 2), although the reason for this phenomenon was unclear. These experiments were repeated three times and similar results were obtained in each experiment.

### 2.2. Effects of the ALSV Vaccine in Preventing Broad Bean Mosaic Disease

**Figure 3:**
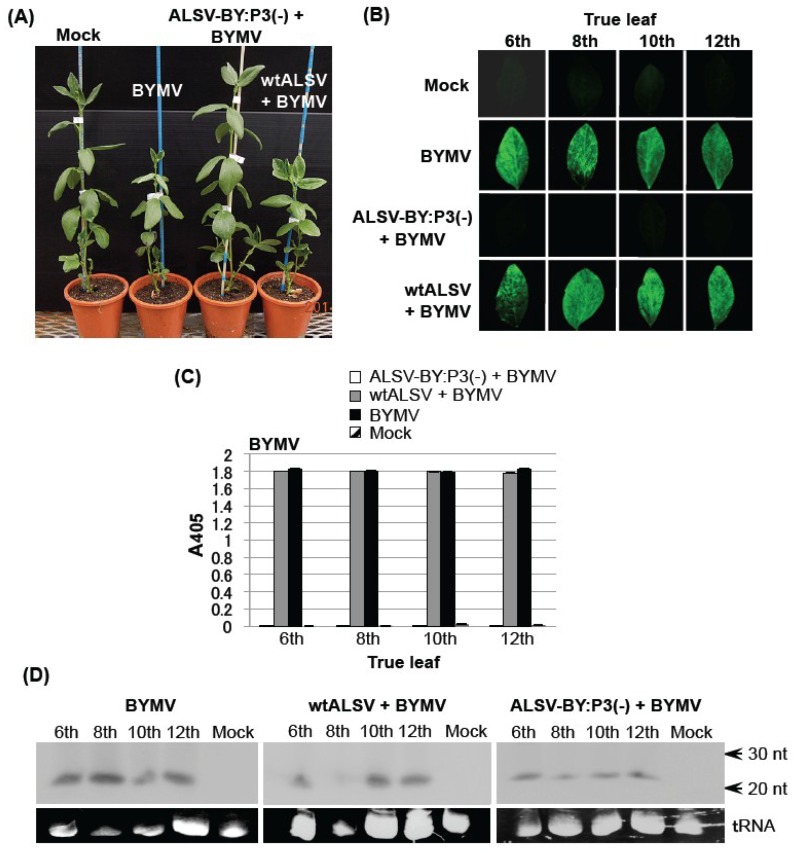
Protective effects of pre-inoculating broad bean plants with an *Apple latent spherical virus* (ALSV) vaccine (ALSV-BY:P3(−)) on challenge inoculation with a *Bean yellow mosaic virus* (BYMV) expressing green fluorescent protein (GFP). (**A**) Symptoms of broad bean plants inoculated with BYMV only, co-inoculated with the ALSV vaccine and BYMV, or wtALSV and BYMV at 21 days post-inoculation (dpi); (**B**) Fluorescence microscopy of the sixth to 12th true leaves of broad bean plants co-inoculated with ALSV-BY:P3(−) and BYMV; (**C**) Enzyme-linked immunosorbent assay (ELISA) measuring BYMV accumulation in the sixth to 12th leaves of the broad bean shown in (**B**); Leaf samples were collected at 21 dpi with BYMV. In both ALSV vaccine + BYMV and wtALSV + BYMV plots, ELISA values of ALSV in the sixth to 12th leaves were approximately 1.8 (data not shown); (**D**) Northern blot analysis of the small interfering RNAs derived from the BYMV-P3 regions of the sixth to 12th leaves of broad bean plants inoculated with BYMV only, co-inoculated with wtALSV and BYMV, or co-inoculated with the ALSV vaccine and BYMV.

The first and second leaves of three-leaf-stage broad bean seedlings were simultaneously inoculated with ALSV-BY:P3(−) and BYMV. In the control BYMV group, chlorotic symptoms and leaf deformation were observed initially on the sixth leaf 7 dpi. Severe mosaic disease, leaf deformation, and leaf necrosis were observed in the seventh to the 12th leaves at 21 dpi. In addition, growth of the whole plants was stunted (Figure 3A) and plants showed leaf necrosis resulting in plant death. Similar symptoms were observed in plants in the wtALSV+BYMV group. In the ALSV-BY:P3(−) + BYMV group, however, no symptoms were observed at any leaf position and no stunting of the stem was found in any plants for two months (Figure 3A).

GFP fluorescence in the sixth, eigth, 10th, and 12th leaves, which showed disease symptoms, was examined 21 dpi of BYMV. In the BYMV and wtALSV + BYMV groups, GFP fluorescence was observed on all leaves examined (Figure 3B). In contrast, no fluorescence was observed in the ALSV-BY:P3(−) + BYMV group, indicating strong suppression of BYMV replication (Figure 3B).

BYMV accumulation was investigated by ELISA at 21 dpi, and high BYMV production was found from the sixth to the 12th leaves in the BYMV and wtALSV+BYMV groups (Figure 3C). In the ALSV-BY:P3(−) + BYMV group, however, no BYMV accumulation was found from the sixth to the 12th leaves (Figure 3C).

Low molecular weight RNA was extracted from the sixth, eighth, 10th, and 12th leaves of the BYMV, wtALSV + BYMV, and ALSV-BY:P3(−) + BYMV groups, and northern blotting was performed, using RNA complementary to the P3 region of the BYMV genome as a probe [8]. siRNAs derived from BYMV-RNA were found in all leaves of the BYMV, wtALSV + BYMV, and ALSV-BY:P3(−) + BYMV groups (Figure 3D).

### 2.3. Effect of the ALSV Vaccine in Preventing Eustoma Mosaic Disease

**Figure 4:**
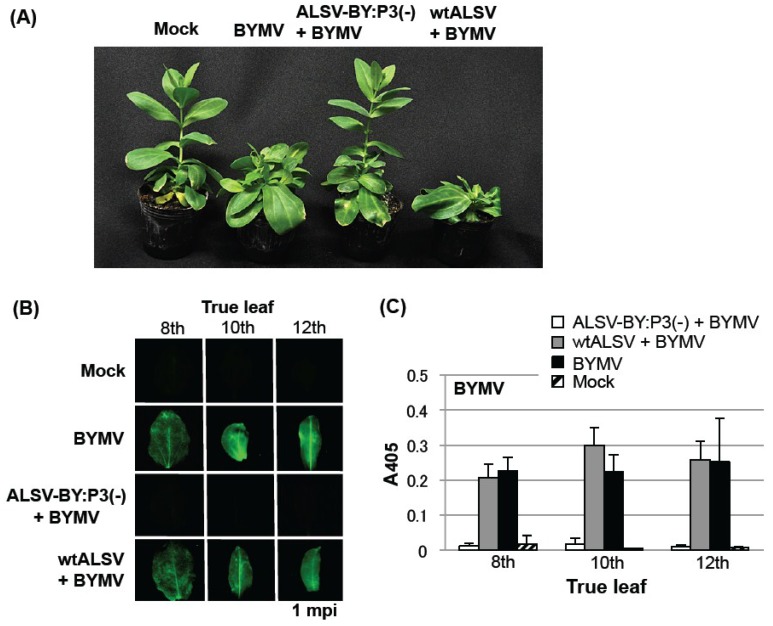
Preventive effect of pre-inoculating eustoma plants with an *Apple latent spherical virus* (ALSV) vaccine (ALSV-BY:P3(−)) on challenge inoculation with a *Bean yellow mosaic virus* (BYMV) expressing green fluorescent protein. (**A**) Symptoms of eustoma plants inoculated with BYMV only, co-inoculated with ALSV vaccine and BYMV, or co-inoculated with wtALSV and BYMV, at two months post-inoculation (mpi); (**B**) Fluorescence microscopy of the eighth to 12th true leaves of inoculated eustoma plants at 1 mpi; (**C**) Enzyme-linked immunosorbent assay (ELISA) measuring the accumulation of BYMV in the sixth to 12th leaves of the eustoma plants shown in (**B**). In both the ALSV vaccine + BYMV and wtALSV + BYMV groups, ELISA values of ALSV in the eighth to 12th leaves were 1.0 to 1.2.

In eustoma plants inoculated with BYMV only (BYMV group) and with wtALSV and BYMV simultaneously (wtALSV + BYMV group), mosaic symptoms began to appear at the base of the fourthth leaf at two to three weeks post-inoculation. Deformation and stunting of the upper leaves above the fourth leaf was observed at one month post-inoculation (mpi), and the whole stems were stunted. At two months post-inoculation, internodal elongation showed marked suppression in comparison to a healthy group (Figure 4A). In plants inoculated with the ALSV vaccine (ALSV-BY:P3(−) + BYMV group), however, BYMV symptoms did not appear even after two months, and subsequent growth was comparable to the mock-treated group (Figure 4A).

**Figure 5:**
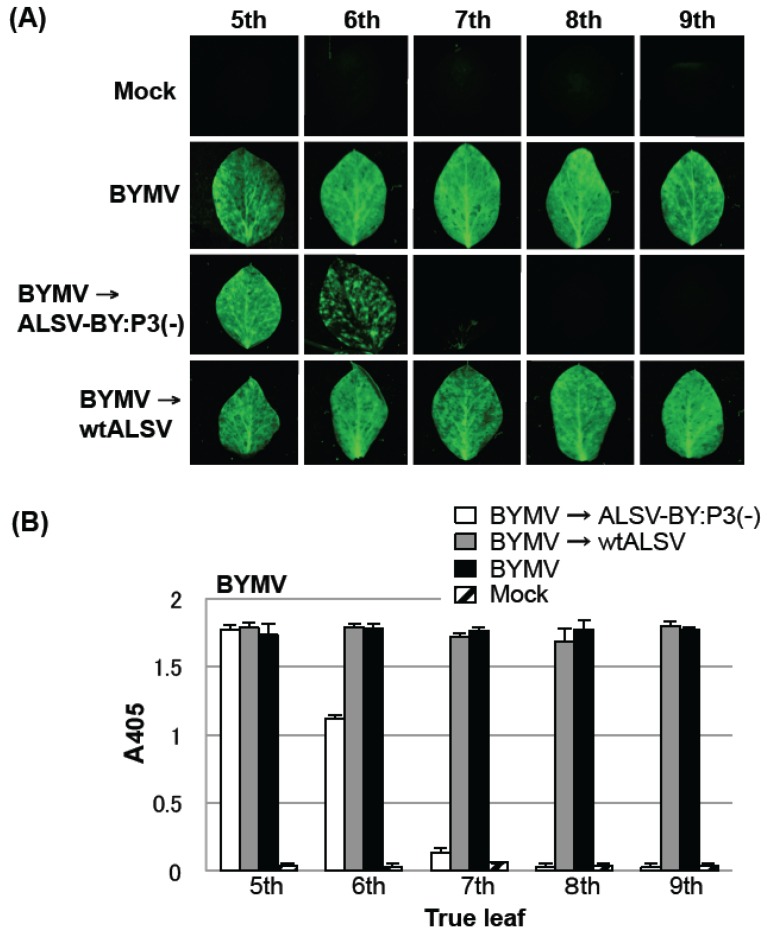
Curative effect of post-inoculation with an *Apple latent spherical virus* (ALSV) vaccine (ALSV-BY:P3(−)) on *Bean yellow mosaic virus* (BYMV) multiplication in mosaic-diseased pea plants infected with BYMV expressing green fluorescent protein. (**A**) Fluorescence microscopy of the fifth to ninth true leaves of a pea plant. The first and second true leaves were inoculated with BYMV and the third and fourth true leaves were inoculated with the ALSV vaccine or with wtALSV. The experimental BYMV series shows plants not inoculated with ALSV vaccine or wtALSV; (**B**) Enzyme-linked immunosorbent assay (ELISA) measurement of BYMV accumulation in pea leaves pre-inoculated with BYMV, followed by post-inoculation with the ALSV vaccine or wtALSV. In both the BYMV→ALSV-BY:P3(−) and BYMV→wtALSV groups, ELISA values of ALSV in the fifth to ninth leaves were 1.2 to 1.8. Samples were collected at 12 days post-inoculation with the ALSV vaccine. The experimental BYMV plot shows plants that were not inoculated with ALSV vaccine or wtALSV. Five plants per each group were used in ELISA test.

GFP fluorescence in the eighth, 10th, and 12th leaves was examined at 1 mpi. GFP fluorescence indicated that BYMV multiplication was distributed in all leaves in the BYMV and wtALSV + BYMV groups (Figure 4B). In contrast, no fluorescence was observed in the ALSV-BY:P3(−) + BYMV group, showing that BYMV multiplication was strongly suppressed (Figure 4B). Accumulation of BYMV and ALSV was investigated in ELISAs. BYMV had accumulated in the BYMV and wtALSV + BYMV groups, but not in the ALSV-BY:P3(−) + BYMV group, indicating marked suppression of viral propagation (Figure 5C). The amount of ALSV detected was similar in plants inoculated with the ALSV vaccine and in plants inoculated with wtALSV.

### 2.4. Curative Effects of the ALSV Vaccine against Mosaic Diseases of Pea and Eustoma Plants

To investigate the curative effects of the ALSV vector vaccine, the first and second leaves of three-leaf pea seedlings were first inoculated with BYMV, and after five days, the third and fourth leaves were inoculated with the ALSV vaccine. In the BYMV and BYMV→wtALSV groups, the fourth and fifth leaves showed faint mosaic symptoms at 5 dpi of ALSV, and there were clear mosaic symptoms from the fifth to the eighth leaves after eight days (data not shown). In the BYMV→ALSV-BY:P3(−) group (pre-inoculation with BYMV, post-inoculation with ALSV-BY:P3(−)), however, mosaic symptoms were seen in the fifth leaf, were weak in the sixth leaf, and were not observed on upper leaves above the seventh leaf (data not shown).

GFP fluorescence in the fifth to ninth leaves was examined 12 dpi of BYMV. In the BYMV and BYMV→wtALSV groups, GFP fluorescence was observed in all leaves examined (Figure 5A). In the BYMV→ALSV-BY:P3(−) group, GFP fluorescence was detected in the fifth leaf, was weak on the sixth leaf, and was not detected at all on upper leaves above the seventh leaf (Figure 5A). Accumulation of BYMV and ALSV in the fifth to ninth leaf was visualized by fluorescence microscopy and quantified by a direct ELISA. Accumulation of BYMV was found in all leaf positions in the BYMV and BYMV→wtALSV groups (Figure 5B). In the BYMV→ALSV-BY:P3(−) group, BYMV accumulation in the fifth leaf was comparable to that observed in the BYMV and BYMV→wtALSV groups. However, BYMV accumulation in the sixth leaf had decreased to approximately two-thirds of the level observed in the BYMV and BYMV→wtALSV groups and was decreased by ~90% in the seventh leaf. BYMV accumulation was not found in the eighth or ninth leaves (Figure 5B). These experiments were repeated three times and similar curative effects were observed in each experiment.

**Figure 6:**
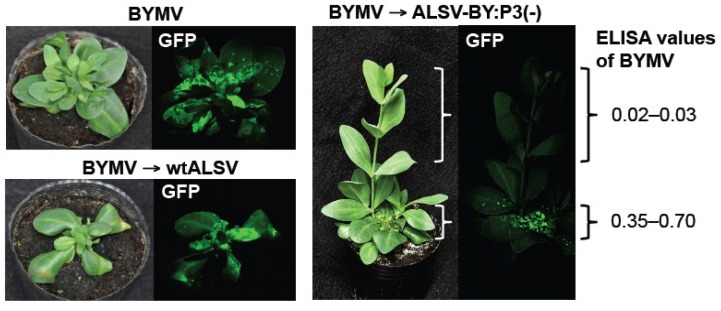
Curative effect of post-inoculation with an *Apple latent spherical virus* (ALSV) vaccine (ALSV-BY:P3(−)) on *Bean yellow mosaic virus* (BYMV) multiplication in mosaic-diseased eustoma plants infected with BYMV expressing green fluorescent protein (GFP). Pictures were taken at one month post-inoculation with the ALSV vaccine. In both the BYMV and BYMV→wtALSV groups, plants showed severe stunting and robust GFP fluorescence. In contrast, in the BYMV→ALSV-BY:P3(−) group, leaves that developed after inoculation with ALSV vaccine showed normal growth and did not support BYMV multiplication.

BYMV-infected eustoma plants were post-inoculated with the ALSV vaccine to investigate whether mosaic disease could be cured. BYMV was inoculated on the first and second pairs of leaves of eustoma seedlings having three pairs of leaves using a particle gun, and plants showing stunting after approximately three weeks were inoculated with ALSV vaccine. In the BYMV group, internodal elongation was halted in the majority of plants, and stem stunting and leaf deformation was found (Figure 6). In the group with the secondary wtALSV inoculation (BYMV→wtALSV group), the stems remained stunted and there was no restoration of growth (Figure 6). In the BYMV→ALSV-BY:P3(−) group, however, at two weeks after ALSV vector vaccine inoculation, new shoots emerging from the stunted stem began normal internodal elongation, and no mosaic disease or deformities were observed in their leaves (Figure 6).

Plants were examined by fluorescence microscopy at 1 mpi with the ALSV vaccine. In the plants of the BYMV and BYMV→wtALSV groups, GFP fluorescence was observed in the leaves with mosaic symptoms and stunting. In the BYMV→ALSV-BY:P3(−) group, however, GFP fluorescence was observed only on the leaves showing stunting or deformation; on the growth that emerged following ALSV vaccine inoculation, internodal elongation had recovered, no mosaic symptoms or stunting were observed, and no GFP fluorescence was detected from the leaves of the new shoots (Figure 6). Leaves in which no GFP fluorescence was observed were tested in ELISAs, and no BYMV accumulation was found (Figure 6). These plants normally grew without showing mosaic and stunting symptoms.

## 3. Discussion

We previously demonstrated the possibility that an ALSV vector harboring part of the sequence of a target viral genome (an ALSV vaccine) can be used as a viral vaccine to inhibit cucumber mosaic disease caused by ZYMV or CMV, as well as spotted wilt of eustoma caused by *Tomato spotted wilt virus* (TSWV) [8,21]. Experiments with *N. benthamiana* have shown that ALSV vector vaccines exert a powerful interference effect against tospoviruses such as *Impatiens necrotic spot virus* and* Iris yellow spot virus* in addition to TSWV [21]. The target virus in the present study was BYMV, which is a pathogenic virus that causes mosaic symptoms in legumes and ornamental flowers worldwide [24]. If ALSV vaccines are effective in preventing different kinds of mosaic disease, they could be used as effective biological pesticides.

As shown in Figure 1 and Figure 2, when peas were pre-inoculated with the ALSV vaccine and then challenge inoculated with BYMV, BYMV could multiply in the inoculated leaf (the fourth leaf), but BYMV multiplication in leaf positions above the fifth leaf was inhibited. This is probably because when ALSV vaccine was inoculated on the first and second leaves, the vaccine first infected the fifth leaf; consequently, BYMV multiplication occurred in the fourth leaf, as infection with the ALSV vaccine was not established. BYMV multiplication was completely inhibited in the upper leaves above the fifth leaf, so that once infection with ALSV vaccine was established in a leaf, a strong defense system was induced. This defense remained fully intact throughout the experimental period. These results indicate that the previously reported effects of the ALSV vector vaccine in protecting against BYMV in the experimental plant *N. benthamiana* [8] are reproducible in the pea plant.

Studies on cross-protection of plant viruses have shown that interference effects are enhanced with an increasing interval between pre-inoculation with a virus (mild strain) and challenge inoculation with second virus (severe strain) [32,33]. This is because infection by a mild strain must become established as a necessary condition for inducing effective cross-protection. In the case of the ALSV vaccine, however, a protective effect is found even when the pathogenic virus is inoculated simultaneously with the vector vaccine [8]. In the present study as well, a strong preventative effect against BYMV was observed in broad bean (Figure 3) and eustoma plants (Figure 4) inoculated simultaneously with the ALSV vaccine and BYMV, and no BYMV symptoms were found and BYMV multiplication was inhibited. We have discussed the reason for this result in a previous study [8], and it is likely that ALSV rapidly invades the shoot apical meristem and establishes a defense system by inducing RNA silencing. For example, we reported that when cucumbers are treated with a mixed inoculation of ALSV and ZYMV, ALSV is found in the apical meristem whereas ZYMV does not invade this tissue [8]. In separate studies, we simultaneously inoculated *N. benthamiana* with ALSV and BYMV and analyzed the plant through* in situ* hybridization with RNA probes against both viruses, and while ALSV was found in the shoot apical meristem, BYMV did not invade this tissue [34].

The ALSV vaccine was particularly effective when used to inoculate a diseased plant, exhibiting a curative effect in virus-infected plants. In the present study, pea and eustoma plants post-inoculated with the ALSV vaccine showed gradual inhibition of BYMV multiplication in their upper leaves (Figure 5 and Figure 6) and BYMV symptoms subsided (Figure 6). In general, it is very difficult to cure plants that are infected with a viral disease and infections should be controlled by taking preventative measures to prevent infection. Once a viral disease has broken out, it is difficult to rescue the plants and usually no treatment is performed. For this reason, in plants such as fruit trees where an individual plant is continuously cultivated over many decades, infection with a viral disease can result in enormous damage. A typical example is observed with the *Plum pox virus* (PPV), which has caused tremendous damage to stone fruits in many different countries, particularly in Europe [35,36]. A PPV outbreak was found in Japanese apricots in Japan in 2009, and despite repeated felling of infected trees, the spread of infection has not been halted. PPV belongs to the genus *Potyvirus*, like BYMV and ZYMV, so an ALSV vector vaccine can be expected to be effective against it. ALSV offers the possibility of curing trees that are infected with PPV.

## 4. Materials and Methods

### 4.1. Plant Material

*N. benthamiana* plants were used for propagating ALSV and BYMV. Pea (*Pisum sativum* cv. Matsushima Kinusaya), broad bean (*Vicia faba* cv. Kawachi Issun), and eustoma (*Eustoma exaltatum* cv. Shalala blue) plants were used to test the interference effects of the ALSV vector vaccine.

### 4.2. ALSV Vaccine and BYMV

The ALSV vaccine against BYMV, ALSV-BY:P3(−), was constructed by inserting the 240-bp P3 coding region (bases 3059 to 3298) of the BYMV genome (GenBank accession No. AB079887) into the 3’-untranslated region of ALSV-RNA2 [8]. This vaccine has been previously shown to elicit preventative effects against BYMV in *N. benthamiana* [8]. The challenge virus used was an infectious cDNA clone of BYMV expressing green fluorescent protein (Ibg-GFP), as described previously [37]. Eustoma plants were inoculated with the cDNA clone using a particle gun, as described below, while pea and broad bean plants were inoculated using a crude extract from Ibg-GFP-infected *N. benthamiana*.

### 4.3. Preparation of ALSV Inocula

Leaves (10 g) of *N. benthamiana* plants were homogenized with 3 volumes of extraction buffer (0.1 M Tris-HCl, 0.1 M NaCl, 5 mM MgCl_2_, pH 7.8) in a Waring blender at 2 weeks post-inoculation with either ALSV-BY:P3(−) or wild-type ALSV (wtALSV). The homogenate was centrifuged at 9000 × *g* for 10 min, and the resultant supernatant was clarified by adding bentonite solution (30 mg/mL) dropwise until it turned transparent yellow in color. Polyethylene glycol 6000 was added to the supernatant to a final concentration of 8% (w/v), and the solution was shaken for 1 h and then centrifuged at 9000 × *g* for 10 min at 4 °C [2]. The precipitate thus obtained was resuspended in 0.2–0.4 mL extraction buffer to give a concentrated ALSV preparation.

RNA was extracted from a concentrated ALSV preparation as follows. An ALSV preparation (50 µL) was vortexed with 150 µL sterile water, 100 µL water-saturated phenol, and 100 µL chloroform and then centrifuged at 10,000 × *g* for 15 min at 4 °C. RNA was precipitated by adding 20 µL 3 M sodium acetate (pH 5.2) and 500 µL 99.5% ethanol to 200 µL of the supernatant. The precipitates were washed in 70% ethanol and resuspended in 10–30 µL sterile water. The RNA concentration was measured using a NanoDrop 1000 spectrophotometer (Thermo Fisher Scientific K.K., Yokohama, Japan), and a 1-µg/µL solution was prepared to yield a concentrated RNA preparation. In this manner, IbG-GFP RNA was also extracted from *Escherichia coli* and prepared at a concentration of 1 µg/µL for use as an inoculum [37].

### 4.4. Viral Inoculation

To investigate the preventative effects of the ALSV vaccine, pea plants were pre-inoculated with the vaccine by rub-inoculating a concentrated ALSV preparation (10 µL per leaf) on the 1st and 2nd leaves of pea seedlings (3-leaf stage), using the carborundum method (Figure 1A). A crude BYMV extract of IbG-GFP-inoculated *N. benthamiana* was prepared by homogenizing leaves in 3 volumes of 0.1 M extraction buffer (pH 7.8), and this crude extract was used for challenge inoculations by the carborundum method on the 3rd and 4th leaves of seedlings that had grown to the 5-leaf stage.

To investigate the curative effect of the ALSV vaccine, crude BYMV extract was first inoculated on the 1st and 2nd leaves of pea seedlings (3-leaf stage). After 5 days, the concentrated ALSV vaccine preparation was rub-inoculated on the 3rd and 4th leaves. The inoculated peas were grown at 25 °C under long-day conditions (16:8 h light:dark photoperiod) and the interference effect was analyzed.

With broad bean plants, a concentrated ALSV preparation and crude BYMV extract were mixed in equal volumes and rub-inoculated on the 1st and 2nd leaves of 3-leaf stage seedlings. The inoculated broad beans were grown at 25 °C under long-day conditions.

With Eustoma plants, inoculation was performed by the particle gun method as described by Yamagishi* et al.* [38], using the Helios Gene Gun system (Bio-Rad, CA, USA). Using eustoma seedlings of 6th-leaf stage (3 pairs of leaves), the 1st and 2nd leaves were inoculated with a concentrated RNA preparation and the 3rd and 4th leaves were inoculated with Ibg-GFP. Each inoculation was performed with 1 shot at 200 pounds per square inch. After the inoculations, plants were maintained in the dark overnight with sufficient humidity and then grown at 25 °C under long-day conditions.

In experiments to investigate the curative effects of the ALSV vaccine, the 1st to 4th leaves of eustoma seedlings were inoculated with Ibg-GFP using the particle gun method as above. At 3 weeks post-inoculation, the 9th to 12th leaves were rub-inoculated with a concentrated ALSV vaccine preparation using the carborundum method. The inoculated plants were grown at 25 °C under long-day conditions. In inoculation tests, 5 plants were used in each test and all the inoculation tests described above were repeated at least 3 times.

### 4.5. Virus Detection

Inoculated leaves were analyzed by western blotting using an antiserum against ALSV approximately 1 week following the ALSV vaccination to confirm infection with the vector. GFP fluorescence, indicating an increase in BYMV production, was detected using a VB-G25 fluorescence microscope (KEYENCE, Osaka, Japan) [8]. Digital images were captured using a Nikon D70 camera. Accumulation of the ALSV vaccine and BYMV were estimated using a direct ELISA with the antibodies for each virus, as described [8]. The BYMV antibody was purchased from the Japan Plant Protection Association (Tokyo, Japan).
